# Granger Causality Analysis of Steady-State Electroencephalographic Signals during Propofol-Induced Anaesthesia

**DOI:** 10.1371/journal.pone.0029072

**Published:** 2012-01-05

**Authors:** Adam B. Barrett, Michael Murphy, Marie-Aurélie Bruno, Quentin Noirhomme, Mélanie Boly, Steven Laureys, Anil K. Seth

**Affiliations:** 1 Sackler Centre for Consciousness Science, Department of Informatics, University of Sussex, Brighton, United Kingdom; 2 Department of Psychiatry, University of Wisconsin-Madison, Madison, Wisconsin, United States of America; 3 Coma Science Group, Cyclotron Research Centre, Neurology Department, University of Liège, Liège, Belgium; Cuban Neuroscience Center, Cuba

## Abstract

Changes in conscious level have been associated with changes in dynamical integration and segregation among distributed brain regions. Recent theoretical developments emphasize changes in directed functional (i.e., causal) connectivity as reflected in quantities such as ‘integrated information’ and ‘causal density’. Here we develop and illustrate a rigorous methodology for assessing causal connectivity from electroencephalographic (EEG) signals using Granger causality (GC). Our method addresses the challenges of non-stationarity and bias by dividing data into short segments and applying permutation analysis. We apply the method to EEG data obtained from subjects undergoing propofol-induced anaesthesia, with signals source-localized to the anterior and posterior cingulate cortices. We found significant increases in bidirectional GC in most subjects during loss-of-consciousness, especially in the beta and gamma frequency ranges. Corroborating a previous analysis we also found increases in synchrony in these ranges; importantly, the Granger causality analysis showed higher inter-subject consistency than the synchrony analysis. Finally, we validate our method using simulated data generated from a model for which GC values can be analytically derived. In summary, our findings advance the methodology of Granger causality analysis of EEG data and carry implications for integrated information and causal density theories of consciousness.

## Introduction

An important challenge for cognitive neuroscience is to characterize directed functional (i.e., causal) connectivity between brain regions, either in the absence of identifiable behaviour or during task performance. In particular, characterizing causal connectivity patterns across different conscious levels (e.g., sleep, anaesthesia, normal wakefulness) is likely to shed important new light on the neural mechanisms underlying consciousness, and may also provide new clinical methods for assessment of intraoperative anaesthetic depth [1]. For example, two potential measures of conscious level predicated on causal interactions among neural elements are ‘integrated information’ (or 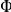) [2] and causal density [3].

One powerful approach to identifying causal connectivity from time series data, originally developed in the 1960s by Norbert Wiener and Clive Granger, is ‘Granger causality’ (GC) [4], [5]. GC embodies a data-driven, statistical time series approach to causal inference based on prediction. The GC from one signal 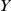 to another signal 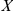 quantifies the extent to which the past of 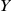 contains information that helps predict the future of 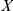 more accurately than when using only the past of 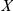. GC is theoretically well founded, is easy to apply when implemented via linear autoregressive modelling, and partly for these reasons has enjoyed accelerating application in neuroscience and beyond [6], [7], [8]. However, despite this growing popularity, rigorous application to neuronal time series data remains challenging for several reasons. First, assumptions of stationarity (requiring in the weak sense constant mean and variance) are often only weakly met in empirical data. Second, common preprocessing steps such as bandpass filtering can interact problematically with GC analysis [9]. Third, comparisons of GC between different conditions can be confounded by bias in the statistical sample since, in finite sample, GC is by definition positive. Here we address these challenges in the context of analysis of steady-state electroencephalographic (EEG) signals. We describe a rigorous analysis pipeline which takes into account potential non-stationarity by applying GC to short data segments that are approximately stationary, and allowing GC to vary across segments. This approach enables us to move beyond the detection of significant causal connections between time series, allowing analysis of the distribution of GC values across segments, and, moreover, inference on differences in distributions of GC values between different steady states. Our method also incorporates a permutation analysis to eliminate statistical bias. We further distinguish our approach by validation against a simulation model for which GC values can be analytically derived.

**Figure 1:**
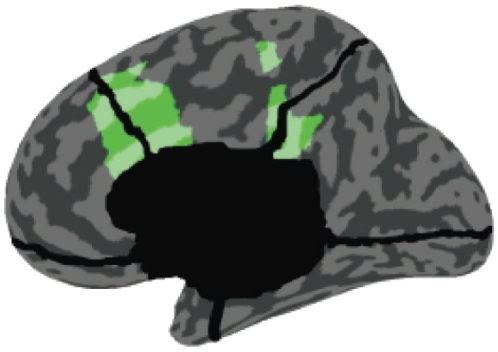
The anatomical locations of the source-localized regions analysed in this paper and in [**11**]. The frontal region (left) is a portion of the anterior cingulate cortex (with extension to the mesiofrontal cortex) and the posterior region (right) is a portion of the posterior cingulate cortex (with extension to the precuneus). Reproduced with permission from Ref. [11].

We illustrate our method by application to high-density, steady-state, source-localized EEG data acquired from subjects during wakeful resting (WR) and when undergoing propofol-induced general anaesthesia (loss-of-consciousness, LOC). Extending a previous analysis, we focused on time series localized to the anterior and posterior cingulate cortices (ACC and PCC respectively), see Figure 1; (ACC coverage extends to the mesiofrontal cortex and PCC to the precuneus). These areas form part of an anatomically-defined ‘mesial highway’ implicated in slow-wave propagation during both anaesthesia and sleep [10], [11]. In the previous analysis, both ACC and PCC showed large increases in gamma (25–40 Hz) power during LOC [11]. Significantly, functional connectivity in the gamma range between these regions also increased during LOC, as measured by phase synchrony. Here, we extend this analysis by examining changes in power and phase synchrony across multiple frequency bands (delta 0.5–4 Hz, theta 4–8 Hz, alpha 8–12 Hz, beta 12–25 Hz, and gamma 25–40 Hz) on a subject-by-subject basis. Our main extension remains however to examine bidirectional GC changes for each subject using a rigorous and well-validated analysis pipeline.

## Methods

### Ethics statement

The data analysed in this study were obtained from a previous study [11] with procedures approved by the Ethics Committee of the Faculty of Medicine of the University of Liège.

### EEG data acquisition and preprocessing

We re-analyzed a subset of the data comprising 5 min of spontaneous high-density EEG recordings sampled at 1000 Hz from each of 7 subjects during both WR and LOC, with LOC defined as clinical unconsciousness (no response to command, Ramsay scale score 5) [12]. LOC was induced via administration of propofol, an intravenous anaesthetic that is widely used in surgical settings which reversibly induces a state of diminished responsiveness behaviourally similar to non-rapid eye movement sleep [13]. Average arterial blood concentrations of propofol were 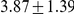 mcg/mL for LOC [11]. Sensor-space EEG data were source modelled using GeoSource (see [11]) and time-courses corresponding to the ACC and PCC regions were extracted. Each region furnished 9 time series, which we averaged to obtain a single time series pair for each subject, in each of the WR and LOC states. From each pair we selected 1–3 non-overlapping artifact-free epochs of variable length. For ease of analysis, for a given subject, the total length of data analyzed in each condition was the same. Across different subjects, the data retained ranged between 140 sec and 200 sec. We paid particular attention to preprocessing steps given the sensitivity of GC to standard manipulations [8], [7]. For the GC analysis we applied the following additional preprocessing steps. Following [9], for each epoch we applied two-way least-squares finite impulse response (FIR) notch filters (49–51 Hz and 99–101 Hz) to remove the 50 Hz mains-electricity line-noise as well as its harmonic at 100 Hz (if left in, these artifacts lead to nonstationarity). Then we downsampled the data to 250 Hz in order to ensure a reasonable model order for autoregressive modelling (see the section ‘Granger causality’ and [14], [8]). Note that higher harmonics of the line noise were rendered higher than the Nyquist frequency following downsampling. No other filtering was carried out; other artifacts were dealt with by choosing artifact-free epochs by inspection.

### Granger causality

In this section we rehearse the formalism of Granger causality in the time and frequency domains. Given two wide-sense stationary time series 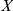 and 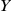 (i.e., time series whose observations have constant means and variances), GC, 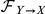, is a measure of the extent to which the past of 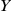 assists in predicting the future of 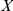, over and above the extent to which the past of 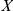 already predicts the future of 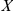 [5]. Standardly, the measure is implemented in terms of linear regressions. Specifically we compare the (unrestricted, Eq. (1), and restricted, Eq. (2)) models
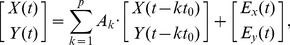
(1)

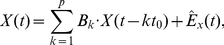
(2)where 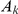 and 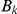 are respectively 2×2 and 1×1 matrices of model coefficients, 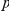 is the model order, 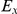, 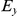 and 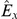 are the residuals, 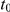 denotes the time between successive observations, and we have assumed for simplicity that 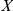 and 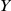 are both zero mean. In practice, 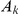 and 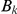, and hence 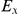, 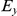 and 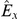 can be derived by standard linear autoregression methods, including ordinary least squares and multivariate Yule-Walker equations [15]. GC is then given by the log-ratio of the variance of the residual in the restricted regression to that in the unrestricted regression:
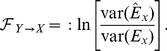
(3)


Importantly, GC has a spectral decomposition that can be used to restrict inferences about causal influence to particular frequency bands [16], [17], [6]. Spectral GC can be thought of as measuring the proportion of power of 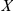 at the given frequency that derives from its interaction with 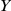. Spectral GC is written in terms of the inverse 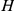 of the transfer matrix 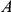, which is defined via the frequency domain representation for the unrestricted regression:
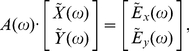
(4)where we use tildes to denote Fourier transform. Explicitly in terms of unrestricted regression coefficients we have
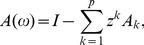
(5)where 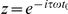, and 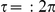 is the circle constant [18]; (see http://tauday.com for several reasons why we adopt 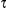 rather than 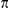 as the circle constant). Let us also introduce the covariance matrix of residuals of the unrestricted regression (1) as
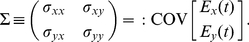
(6)Then the spectral GC at a given frequency 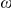 is given by
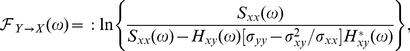
(7)where ‘*’ denotes complex conjugation. The transformation 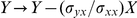 leaves GC invariant, but diagonalizes the covariance matrix 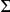 of residuals and simplifies the expression for spectral GC to
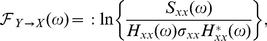
(8)where now we have from Eq. (4) and 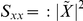 that
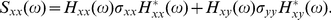
(9)Written this way, we see explicitly how spectral GC is measuring the contribution of causal power relative to intrinsic power.

To obtain the ‘band-limited’ GC for a frequency band defined by the range 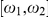, which we denote as 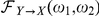, we compute the mean spectral GC across the range, thus
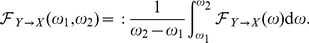
(10)It is noteworthy that the total time-domain GC is given by the mean spectral GC over all frequencies up to the Nyquist frequency 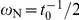:
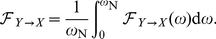
(11)


### Simulation model

To validate our GC methodology, we simulated data from a multivariate autoregressive model with white noise error terms, a model for which we were able to derive true GC values analytically. The general such system is specified by:
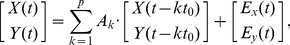
(12)where 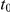 is the time between observations and each 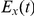 and 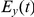 are independent Gaussian random variables of mean 0 and variance 1. True spectral GC values are obtained as follows. Since the residuals are uncorrelated in this model, spectral GC is given by Eq. (8). The transfer matrix is given by (5), and its inverse 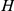 by
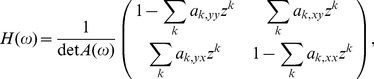
(13)where 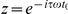. From this, and Eq. (9), and using that the covariance matrix of residuals is the identity, we have
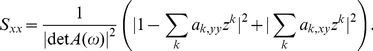
(14)Putting these together into Eq. (8) yields
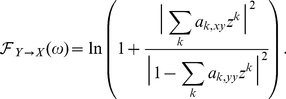
(15)For 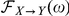, simply swap 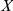 and 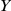 in Eq. (15).

## Results

### Granger causality analysis of EEG data

**Figure 2:**
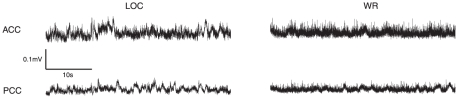
Representative samples of data used in the GC analysis. The data have been notch filtered at 50 Hz and 100 Hz and downsampled to 250 Hz.

**Figure 3:**
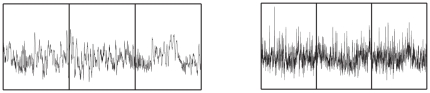
Three representative 2 sec segments used in the GC analysis from the ACC during LOC (left) and WR (right). Vertical lines indicate segment boundaries. The time series shown for each segment have been preprocessed to remove the linear trend and renormalized to each have mean of 0 and standard deviation of 1.

GC analysis was conducted on artifact-free epoched time series, following notch filtering and downsampling, reflecting mean EEG activity within two source-localized brain areas, the ACC and the PCC, recorded from subjects during normal wakeful resting (WR) and under propofol sedation (LOC). Figure 2 shows 40 sec representative samples of data from each area during both LOC and WR. These samples exhibit non-stationary features such as local linear trends at time scales of approximately 1 sec, which would confound GC analysis. To avoid confounds due to nonstationarity we divided the data into approximately stationary non-overlapping short segments from each of which we removed the mean and applied a linear detrend [19], [20]. We chose segment lengths of 2 sec (i.e., 500 time points) in order to strike a balance between stationarity (shorter time series are more likely to be stationary) and model fit (longer time series support better parameter estimation for locally valid linear autoregressive models). For our data, shorter segments (1 sec) led to poor model fitting for many segments, while longer segments (4 sec) were frequently nonstationary; a 2 sec segment length is also in line with previous investigations of stationarity of EEG obtained from sleeping human subjects [21], [22]. Dividing each epoch into non-overlapping 2 sec segments furnished at least 70 segments per condition per subject. Figure 3 illustrates representative segments (with additional normalization of standard deviation for clarity of illustration).

We next computed, for each segment, the recommended model order (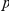 in equations (1) and (2)) as given by the Akaike information criterion (AIC) [23]. (We also computed the Bayesian information criterion [24]; however this criterion often failed to reach a minimum.) The 95th percentile of the values obtained was 20 (corresponding to 80 ms). We used this as our model order throughout the GC analysis.

We next carried out the following GC analysis method for each subject, condition (WR or LOC), direction, and frequency band (delta, theta, alpha, beta, gamma). First, for each 2 sec segment we calculated numerical estimates of GC using Eqs. (1), (2), (8) and (10), using the model order 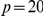, and denoted these by 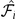, where 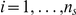, and 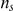 is the number of segments per condition for the given subject. These computations were performed using the Granger Causal Connectivity Analysis toolbox implemented in MATLAB (Natick, MA) [8].

Numerical GC values obtained directly from finite data yield biased estimates of the true GC of the underlying process. In our next step we estimated and removed the bias by applying the following permutation procedure. For each subject, condition, direction and frequency band, we selected 1000 random pairs of 2 sec segments from the ACC with (non-corresponding) 2 sec segments from the PCC. We then computed the numerical GC for each pair, 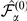, where 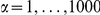. Since each pair has a true GC of zero by definition, the distribution of observed GC values across all pairs approximates the null distribution for zero GC in processes that closely match the analyzed processes. From the empirical null distribution we extracted the mean (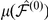) and the standard error (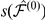, i.e. standard deviation divided by 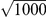). Approximately unbiased GC values were then obtained by subtracting the mean of the null distribution from the biased estimates: 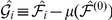. It is worth noting that, for time-domain GC, and a true linear autoregressive process, this de-biasing procedure is asymptotically exact. This follows because estimates of time-domain GC for such processes asymptotically follow a non-central chi-squared distribution with the true GC value given by the non-centrality parameter [16]. Therefore, the above procedure will furnish exactly unbiased GC values if any factors distorting the distribution away from a chi-square distribution affect the null and non-zero true GC distributions in the same way. These factors may include (i) aspects of the data that are not exactly linear autoregressive, (ii) analysis of short segments which challenge accurate model fitting, (iii) analysis in the frequency domain rather than the time-domain (exact distributions are not known for the frequency domain). In practice, even if these factors apply non-uniformly to null and non-null distributions, our debiasing procedure nonetheless provides improved empirical estimates of the true (unbiased) GC values. We further validate our methods by application to a simulation model (see Section ‘Simulation model’).

Finally, for each subject, condition, direction and frequency band, we obtained an estimate of the mean GC, 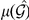 by taking the mean of the approximately unbiased estimates across segments,
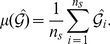
(16)An estimate of the standard error 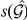 of this estimate is then given by
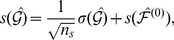
(17)where 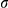 denotes the standard deviation of the 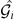.

We repeated the above procedure for the time-domain, with time-domain GC values computed in approximation by taking the mean over frequencies from 0.5–40 Hz (Eq. (10)). We avoided the explicit time-domain GC (3) because that was found to sometimes contain residual spurious contributions from the line noise at 50 Hz [9]; (we took 40 Hz as a safe frequency cut-off for avoiding this, and there was negligible power above 50 Hz in all data). We also repeated the procedure to compute GC values at all integer frequencies from 1 Hz to 40 Hz.

To confirm validity of application of linear regression models of order 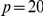 to each of the data segments, following [8] we performed both the Durbin-Watson test for whiteness of residuals [25] and the consistency test of Ding *et al* [26]. Residuals were reliably white across all segments for all subjects (mean 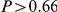), indicating that the linear regression models adequately accounted for the variance in the data. The mean consistency value across segments was 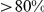 for 4/7 subjects and 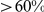 for 6/7 subjects, verifying that the models are capable of regenerating the observed data with high accuracy. Together, these results validate the suitability of GC analysis for the data.

**Figure 4:**
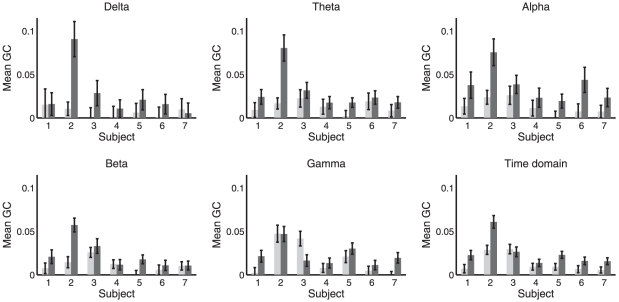
Mean band-limited GC computed using (16) in the direction PCC 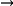
 **ACC, in WR (light) and LOC (dark).** Each panel shows a different frequency band; the bottom-right panel shows the time-domain. Error bars show standard error (17).

**Figure 5:**
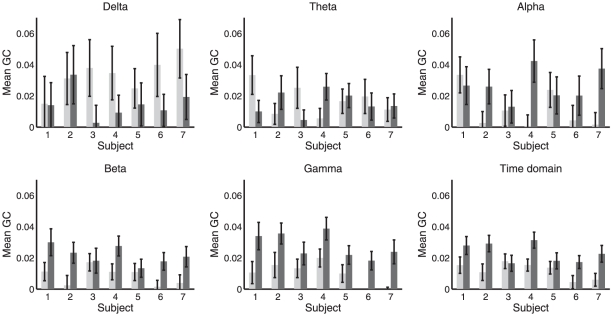
Mean band-limited GC computed using (16) in the direction ACC 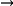
 **PCC, in WR (light) and LOC (dark).** Each panel shows a different frequency band; the bottom-right panel shows the time-domain. Error bars show standard error (17).

**Figure 6:**
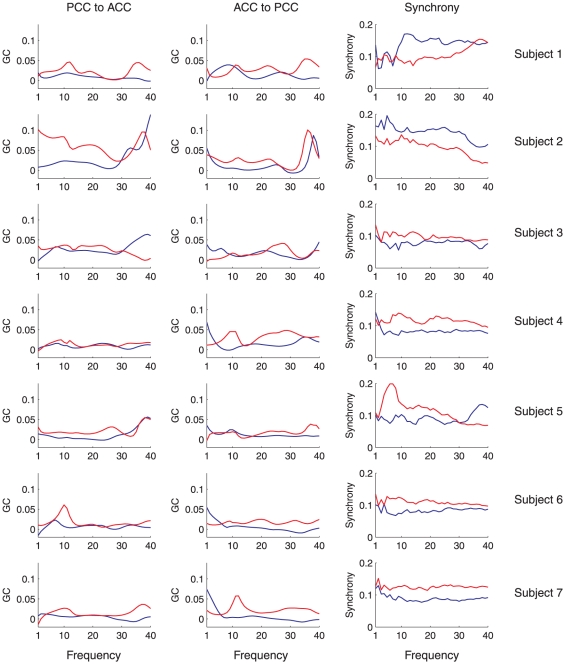
Plots of mean spectral GC and synchrony against frequency. Left column shows the mean spectral GC in the direction PCC
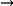
ACC by frequency, for WR (blue) and LOC (red). The middle column shows the same data for mean GC in the direction PCC
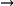
ACC. The right column shows mean phase synchrony by frequency. Each row shows a different subject.

Figures 4 and 5 show band-limited and time domain GC for the directions (PCC
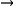
ACC) and (ACC
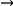
PCC) respectively. Figure 6 shows mean GC (and phase synchrony, see below) at all integer frequencies for each condition and each subject. To assess significance of differences in GC between WR and LOC, for each subject, condition, direction and frequency, we performed a Wilcoxon rank sum test to compare the distributions of 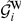 and 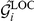, i.e., the distributions across segments of approximately unbiased GC estimates for WR and LOC respectively. (Note that we are interested in whether GC values differ between conditions, not in whether any particular GC value is statistically significant.) The results of these tests are given in Table 1 (PCC
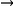
ACC) and Table 2 (ACC
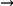
PCC), at various significance levels. Verifying the consistency of these results, in all cases in which a significant difference was found at a false discovery rate of either 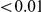 or 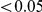, there was no overlap in the corresponding error bars in the corresponding graph (see Figs. 4 and 5). The overall outcome of this analysis is that changes in mean GC from WR to LOC show high inter-subject consistency, with most subjects exhibiting a bidirectional *increase* in GC between the ACC and the PCC during LOC, particularly in the beta and gamma bands.

**Table 1:** Significant changes in GC from WR to LOC, in the direction 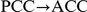, in each frequency band, and also in the time domain.

Subject	1	2	3	4	5	6	7
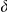	n/s	(+)	n/s	[+]	n/s	[+]	n/s
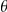	[+]	+	n/s	n/s	+	n/s	n/s
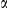	n/s	[+]	n/s	n/s	[+]	n/s	n/s
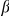	n/s	+	n/s	n/s	+	n/s	n/s
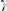	(+)	n/s	[−]	n/s	n/s	n/s	(+)
time domain	(+)	+	n/s	n/s	+	n/s	[+]

‘+’ indicates an increase during LOC, ‘−’ indicates a decrease, and ‘n/s’ indicates no significant change. Absence of brackets indicates significance at a false discovery rate of 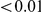, round brackets indicate significance at a false discovery rate of 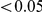, and square brackets indicate significance at the 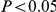 level.

**Table 2:** Significant changes in GC from WR to LOC, in the direction 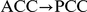, in each frequency band, and also in the time domain.

Subject	1	2	3	4	5	6	7
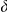	n/s	n/s	n/s	n/s	n/s	n/s	n/s
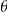	n/s	n/s	n/s	(+)	n/s	n/s	n/s
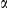	n/s	n/s	n/s	[+]	n/s	n/s	n/s
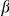	n/s	+	n/s	[+]	n/s	(+)	(+)
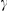	[+]	(+)	n/s	[+]	[+]	+	+
time domain	n/s	+	n/s	[+]	n/s	+	(+)

‘+’ indicates an increase during LOC, ‘−’ indicates a decrease, and ‘n/s’ indicates no significant change. Absence of brackets indicates significance at a false discovery rate of 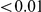, round brackets indicate significance at a false discovery rate of 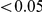, and square brackets indicate significance at the 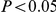 level.

### Granger causality of simulated data

**Figure 7:**
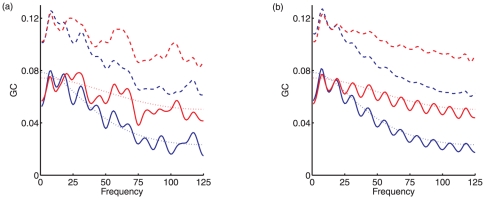
Analytical and numerical GC in a simulation model. (a) Mean spectral GC obtained from 200 sec of simulated data, obtained from the model described by Eqs. (12) and (18), implementing bidirectional causality between two variables 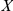 and 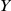. Blue lines show GC in the direction 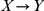; red lines show GC in the opposite direction. Dashed lines show numerical estimates of GC prior to debiasing, solid lines show numerical estimates following debiasing, and dotted lines show the analytical GC values. (b) Mean numerical GC estimates, before and after debiasing, computed across 100 instantiations of the model, with each instantiation generating 200 sec of simulated data.

To validate the GC analysis of the EEG data, we applied the same procedure to the simulation model described in the section ‘Simulation model’, for which analytical GC values could be computed. The model we simulated had the following non-zero regression matrices
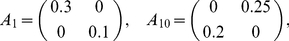
(18)chosen so that there is frequency-dependent bidirectional causality between 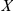 and 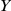. We assumed a sampling rate of 250 Hz, so that 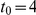 ms. Using this model, we computed bidirectional spectral GC analytically using Eq. (15), and compared results with those obtained from simulated data consisting of 100 segments of length 2 sec each. For comparison with the GC analysis of the EEG data, we used a model order of 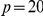. Figure 7 (a) shows the analytical GC and the numerically computed (‘sample’) GC both before and after application of the debiasing method described in the section ‘Granger causality analysis of EEG data’. It is clear that raw sample GC values have a substantial positive bias, which is significantly reduced uniformly across frequencies by the debiasing method. Figure 7 (b) shows the results of repeating this procedure over 100 different instantiations of the model, confirming the effectiveness of the debiasing method. We note that there is some remaining oscillation of the debiased value around the analytic value, but that this oscillation is small. Finally, we performed Wilcoxon tests on numerical GC estimates across 100 segments (of a single instantiation of the model) to look for significant differences in GC in the two directions. We tested for differences in band-limited GC in the frequency bands 1–25 Hz, 26–50 Hz, 51–75 Hz, 75–100 Hz and 100–125 Hz. Consistent with the analytical profile of the spectral GC measure, we found no significant difference in the 1–25 Hz band, 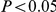 in the 26–50 Hz band, and 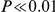 in all the other bands. These observations further attest the consistency of our method.

### Phase synchrony analysis of EEG data

We next compared the results of our GC analysis of the EEG data with a phase synchrony analysis of the same data. Unlike GC, phase synchrony provides an *undirected* measure of functional connectivity. Synchrony measures have also been widely applied in studies of both conscious content and conscious level (see [27] for a review). The present analysis extends the previous analysis [11], which found that on average, across all subjects, phase synchrony increased during LOC in the theta, alpha and gamma frequency bands. Here, augmenting this group level analysis, we investigate changes in synchrony for each subject individually.

As described in the Methods section, in contrast to the GC analysis, for the synchrony analysis we did not notch-filter or downsample the data during preprocessing. (We did repeat the synchrony analysis with downsampling to 250 Hz; the only effect of this was loss of significance for a few cases, due to the throwing away of data. Notch filtering would be redundant because bandpass filters are applied as part of the synchrony computation.) For each data point we computed the instantaneous synchrony between the ACC and PCC in each of the five frequency bands (delta, theta, alpha, beta, gamma) following the spatial analytic phase difference (SAPD) method of [28], which was also the method used in [11]. This procedure works as follows: First the time series from each epoch of data were filtered using two-way least-squares FIR filters, with pass band given by the frequency band under consideration. Next, instantaneous phases were computed for each data point, via Hilbert transform. Phases were then unwrapped, allowing instantaneous differences between phases of the ACC and the PCC to be computed. These instantaneous differences were mapped back onto the interval 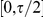 to obtain the SAPD at each time-point. Finally, a binary value of phase synchrony at each time-point was obtained by associating SAPD values below 0.2 radians with synchrony of 1, and SAPD values above 0.2 radians with synchrony of 0. (We repeated our calculations using a continuous measure of phase synchrony, obtained directly from SAPDs; results were unchanged.)

**Figure 8:**
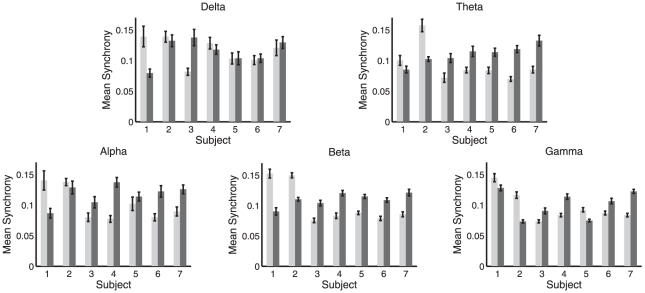
Mean phase synchrony in WR (light) and LOC (dark). Each panel shows a different frequency band. Error bars show standard error. Mean and standard error computed across 10 sec windows of data, see main text.

For each subject and condition we divided the synchrony data into 10 sec non-overlapping windows. For each window we calculated the proportion of time-points with above-threshold (i.e., 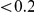 SAPD) phase synchrony within each frequency band. Figure 8 shows the mean of this proportion across all 10 sec windows, in each frequency band, for each subject individually. As for GC, we also computed synchrony at all integer frequencies from 1 Hz to 40 Hz (using pass bands of 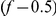 Hz to 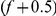 Hz for each frequency 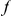). Figure 6 shows synchrony at all frequencies for each condition and subject, furnishing a direct comparison with the GC analysis. Together, Figures 8 and 6 indicate that phase synchrony generally increases during LOC though with less consistency across subjects than as compared to the GC analysis. Supporting this interpretation, Table 3 shows the outcome of significance tests on the difference in phase synchrony between WR and LOC, calculated using the Wilcoxon rank sum test. In contrast to the GC analysis, the results of this analysis show substantial variability between subjects, particularly in the beta and gamma bands, for which two subjects showed a highly significant *decrease* in synchrony during LOC while the majority of subjects showed a highly significant *increase*. Nonetheless, the grand average across all subjects showed an increase during LOC in the theta, alpha, beta and gamma bands and a decrease in the delta band (significance not tested for here), reconfirming the group-average analysis in [11].

**Table 3:** Significant changes in mean phase synchrony from WR to LOC in each frequency band.

Subject	1	2	3	4	5	6	7
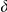	−	n/s	+	n/s	n/s	n/s	n/s
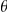	n/s	−	n/s	+	+	+	+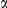	(−)	n/s	n/s	+	n/s	+	+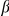	−	−	+	+	+	+	+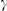	n/s	−	+	+	−	+	+

‘+’ indicates an increase during LOC, ‘−’ indicates a decrease, and ‘n/s’ indicates no significant change. Absence of brackets indicates significance at a false discovery rate of 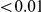, round brackets indicate significance at a false discovery rate of 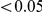, and square brackets indicate significance at the 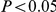 level.

### Power spectral density analysis of EEG data

**Figure 9:**
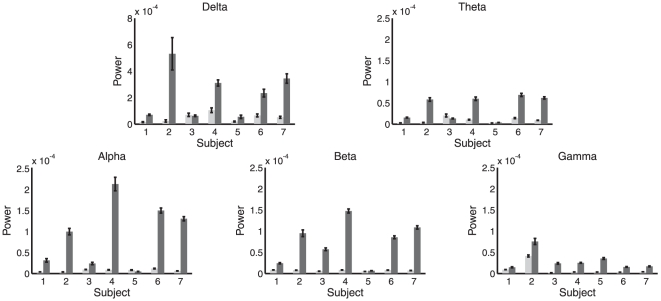
Mean power spectral density in the ACC in WR (light) and LOC (dark). Each panel shows a different frequency band. Error bars show standard error. Mean and standard error computed across 10 sec windows of data, see main text.

**Figure 10:**
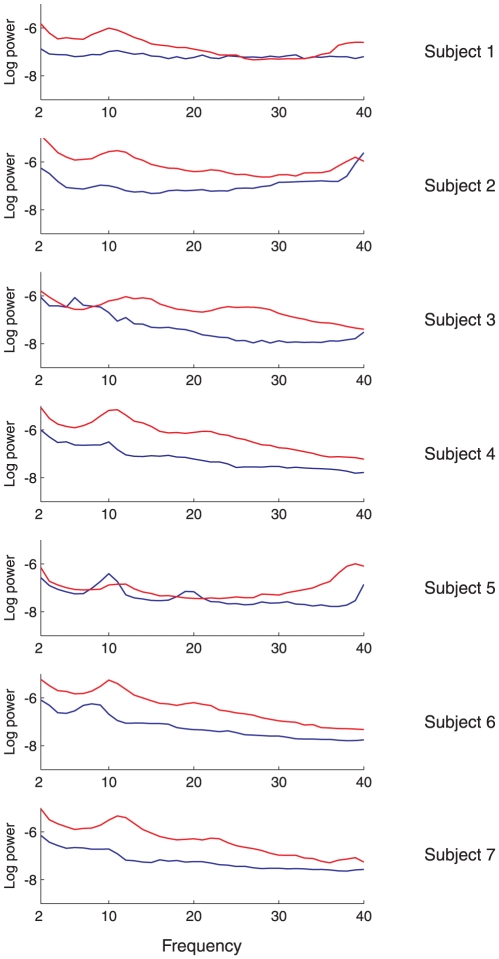
Mean (log) power spectral density in the ACC during WR (blue) and LOC (red) for each subject. Logarithms are to base 10.

To examine changes in spectral power on a subject-by-subject basis, we applied a fast Fourier transform to each of the 10 sec windows identified in the previous synchrony analysis. For each subject and frequency band we computed the mean power spectral density (PSD) across all windows, in both the ACC (Figure 9) and the PCC. Table 4 shows the outcome of Wilcoxon rank sum significance tests on the difference in PSD between LOC and WR for each subject and frequency band. The majority of entries in this table show a significant increase in PSD during LOC, in line with the group-average results described by [11]. Corroborating these findings, Figure 10 shows the full PSD spectra in WR and LOC for the ACC region in each subject, averaged over all the windows.

**Table 4:** Significant changes in mean power from WR to LOC in each frequency band and for both the ACC and the PCC.

Subject	1	2	3	4	5	6	7
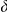 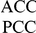	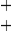	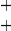	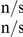	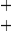	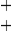	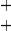	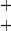
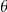 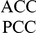	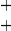	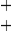	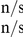	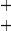	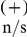	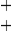	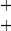
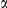 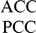	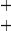	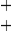	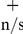	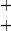	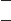	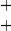	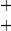
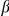 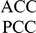	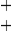	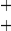	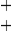	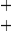	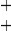	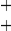	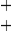
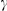 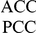	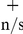	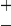	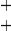	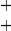	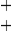	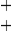	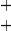

‘+’ indicates an increase during LOC, ‘−’ indicates a decrease, and ‘n/s’ indicates no significant change. Absence of brackets indicates significance at a false discovery rate of 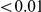, round brackets indicate significance at a false discovery rate of 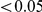, and square brackets indicate significance at the 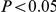 level.

## Discussion

We have presented a method for applying GC analysis to steady state EEG data, that (i) accommodates non-stationarity by dividing the data into short approximately-stationary segments, and (ii) systematically removes bias by permutation analysis. Our method is generally applicable in neuroimaging contexts that generate continuous time series data at sampling rates reflecting neural interactions (magneto/electroencephalographic signals, intracranial recordings, electrocorticographic signals, other local-field-potential signals). We demonstrated the efficacy of our method via a rigorous set of simulations for which GC could be solved analytically. We illustrated its value by application to source-localized steady-state high-density EEG data obtained from healthy human subjects undergoing propofol-induced anaesthesia, examining changes in bidirectional GC between the ACC and PCC as subjects transitioned from wakeful resting (WR) to loss-of-consciousness (LOC). We found an increase in bidirectional GC during LOC that was most pronounced in the beta (12–25 Hz) and gamma (25–40 Hz) frequency bands and which was observed consistently across subjects. A comparison with a phase synchrony analysis (following [11]) showed that the changes in GC were more consistent across subjects than were changes in synchrony. Nonetheless, both GC and phase synchrony pointed to *increased* dynamical connectivity between the ACC and PCC during LOC at the group level. Changes in spectral power were also consistent across subjects, with power in most frequency bands generally increasing during LOC.

### A rigorous methodology for GC analysis

The increasing focus on functional brain networks underlying cognition [29] requires well validated methods for extracting functional connectivity from time series data obtained via neuroimaging. While robust methods exist for identification of undirected functional connectivity (e.g., synchrony, correlation), methods for extracting directed functional (i.e., causal) connectivity are less well established. Among these methods, GC is especially promising because of its simple conceptual and statistical foundations (relative predictive ability and autoregressive modelling, respectively, see [7]), and because it does not require strong priors on the underlying connectivity patterns. (GC can be contrasted with ‘dynamic causal modelling’ [30], which aims at assessing effective connectivity rather than (directed) functional connectivity. Functional connectivity describes directed or undirected network dynamics which need not univocally map onto underlying structural connectivity, whereas effective connectivity aims to infer the underlying physical generative processes [7], [31].) GC also admits a useful interpretation in terms of information transfer because, for Gaussian variables, it is equivalent to transfer entropy [32]. Importantly, changes in GC are not confounded by changes in spectral power: changes in power simply rescale prediction error for both the unrestricted (1) and restricted (2) regressions by the same factor, leaving GC (3) invariant. Despite these advantages, application of GC to empirical data requires great care because GC estimates are readily confounded both by unmet assumptions on the data (e.g., stationarity [20]) and by the impact of standard preprocessing techniques such as bandpass filtering [8], [9]; moreover, GC analysis in sample yields a biased estimate of the ‘true’ GC, complicating comparisons between conditions and subjects. The method described here overcomes these difficulties. In summary, for steady-state EEG data, for comparison of GC values between experimental conditions, we recommend the following steps:

1. Remove any line-noise artifact via notch filtering; avoid bandpass filtering.
2. Choose a minimum timescale for interactions within the system under consideration; downsample the data to a rate reflecting this timescale.
3. Choose a segment length over which the data remain approximately stationary, reflecting a trade-off between increased stationarity (better for short segments) and parameter estimation (better for long segments); partition the data into non-overlapping segments of the chosen length, removing the linear trend and mean from each segment. Exclude segments containing artifacts.
4. For each segment, estimate the model order (e.g., using the Akaike or Bayesian information criterion [23], [24]); compute a high percentile (e.g. 95th) of the recommended model order across all segments.
5. Using this model order, compute GC in both directions for all pairs of variables and for all frequencies of interest. For band-limited GC, integrate spectral GC across the relevant frequencies; for time-domain GC, integrate across all frequencies (up to the Nyquist frequency), omitting any frequencies contaminated by line-noise removal.
6. To estimate the bias in GC values for a particular connection and frequency, compute the mean numerical GC at this frequency between 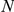 randomized non-corresponding pairs of segments from the predictor and predictee variables (use large 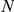, e.g., 1000).
7. Subtract the estimated bias from each raw GC value to obtain an approximately unbiased estimate.
8. Assess significance using a Wilcoxon rank sum test on the distribution of approximately unbiased GC estimates across segments.

Elements of the above method have been proposed previously. Analysis of short time-windows was advocated by Hesse *et al* [20]; however their emphasis was on extracting *time-varying* GC over short time-scales and not on accurate estimation of GC for steady-state data. To our knowledge, the issue of bias has not been examined until now, possibly because most previous studies have been concerned with inference on the statistical significance of individual GC values and not on comparing distributions of GC values across conditions, as we do here. Importantly, we have been able to demonstrate the efficacy of our method with respect to debiasing via our novel analytically solvable model of spectral bidirectional GC.

The context of spectral bidirectional GC between two variables is deliberately simple. The method is however readily extensible to more complicated situations including conditional GC (in which the GC between each pair is conditioned on the common causal influence of other variables, see [6], [9]) and ‘multivariate’ or ‘block’ GC in which causality is assessed between two (or more) multivariate variables (i.e., variables consisting of 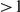 time series) [33]. With respect to preprocessing we have emphasized the need to avoid bandpass filtering. While GC is theoretically invariant to very general filtering, in practice GC estimates are often confounded by increases in empirical model order entailed by the application of a filter [9]. Hence we recommend that filtering be used only where absolutely necessary to ensure stationarity (e.g., application of a notch filter to remove line noise); bandpass filtering should not be applied as a panacea for artifact removal; furthermore, bandpass filtering is entirely inappropriate for estimation of GC within specific frequency ranges. In the latter case, the correct approach is to compute spectral GC at all frequencies and then integrate over the desired range (‘band-limited’ GC, see [9]).

### Dynamical neural correlates of propofol anaesthesia

The neurophysiological changes accompanying propofol-induced LOC have been extensively studied. Alkire and colleagues found using positron emission tomography a reduction in global brain metabolism of about 50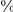 [34]; however global brain metabolism is not a reliable predictor of conscious level, as demonstrated by patients who recover from a vegetative state while still exhibiting dramatically reduced brain metabolism [35]. While a large number of subsequent studies have focused on region-specific neural activity changes during anaesthesia, only recently have researchers studied changes in connectivity. Connectivity studies of propofol-induced LOC have now leveraged multimodal neuroimaging methods including functional magnetic resonance imaging [36], [37], [38], [39], EEG [11], [40], [41], and electrocorticography [42]. Other important studies assessed connectivity changes using different anaesthetic agents including isoflurane and halothane [43] and midazolam [44], the latter in combination with transcranial magnetic stimulation (TMS). A complex picture is emerging from these studies, indicating that anaesthetic LOC is associated with modulated operation of discrete networks rather than global or regional suppression or enhancement of neural activity [45]. However, the diversity of methodologies, potential anaesthetic pathways, and neuroimaging results, together indicate a need for overarching theories specifying unifying dynamical mechanisms.

Addressing this need, a variety of theoretical interpretations have been offered to account for the sedative effects of anaesthetics, including impaired thalamocortical connectivity [43], ‘cognitive unbinding’ of low-level sensory and high-level executive cortical regions [46], [47], reduced information integration [45], [2], [48], and diminished causal density among participating brain regions [49], [3]. The latter two notions specifically involve causal interactions and so are particularly relevant to the present approach. Integrated information uses information theory to capture the extent to which a system considered as a whole generates more information than when considered as a set of independent parts [2], [50], [48]. Causal density uses GC to measure the overall level of causal interactivity sustained by a system [49], [3]. Both measures are motivated by the observation that conscious experiences seem, at the level of phenomenology, to be simultaneously highly differentiated (each experience is different from every other experience) and highly integrated (every experience appears as a unified whole). Both measures also account for experimental observations that consciousness seems to be lost in situations in which the underlying neural dynamics are disintegrated [44], [51] or pathologically integrated, as in generalized epilepsy [52].

The ability to detect directed functional brain networks during anaesthetic LOC is therefore key to refining, as well as differentiating between, the above theories. To our knowledge, only one previous neuroimaging study has attempted this. Lee and colleagues [41] used a method based on asymmetry of modulations of scalp EEG signals, finding diminished feedback connectivity during LOC. However, their method is not widely used as compared to GC and its properties are less well understood. In a related study, Ferrarelli *et al* examined effective connectivity during anaesthesia by perturbing the brain using TMS and observing cortical response patterns [44]. However, this perturbational method does not characterize directed functional networks *per se*. In this context, the method we have described opens the way to explicitly linking theoretically-motivated measures of conscious level with experimentally available data. Our method is also supported by the increased between-subject consistency we observed in modulations of GC, as compared to modulations of phase synchrony, suggesting that GC analysis may offer increased robustness as well as sensitivity to directed interactions.

Stepping back, most theoretical views share the notion that anaesthetic LOC is associated with decreased functional (or effective) connectivity, whether directed or undirected. On the face of it, these views contrast with our results which showed increased GC and phase synchrony during LOC, as well as increased power. However, increased functional connectivity could be consistent with overall reduction of causal density and/or information integration under at least two scenarios. First, high values of some measures (synchrony, bivariate GC) could reflect pathologically increased integration at the expense of differentiation. This view aligns with the increased cortical-subcortical synchrony observed during LOC associated with generalized epilepsy [52]; these authors argue that enhanced synchrony ‘blocks access’ to a neuronal global workspace. Second, in any GC analysis, common unmeasured sources can influence results [53], [29]. In this context, it is important to recognize that the present results are based on analyzing connectivity between only two areas, the ACC and the PCC, which form part of a distinctive anatomical cortical backbone (the ‘mesial highway’) [54]. Thus it is plausible that increased functional connectivity between the ACC and the PCC may reflect a wider disruption or disintegration of functional connectivity, once other regions are taken into account. Adequate tests of theories based on functional disintegration therefore require extending the present analysis to incorporate a broader range of cortical (and possibly subcortical) sources, together with fully multivariate measures of causal connectivity.

### Conclusions

We have described a methodological pipeline for GC analysis of steady-state EEG signals, accommodating nonstationarity, eliminating bias, and validated against an analytically solvable model. This pipeline represents a contribution towards the general problem of identifying directed functional connectivity in brain networks [29], with specific relevance to the problem of measuring conscious level [55], [3]. The bidirectional increases in GC that we observed between the ACC and PCC appear to challenge current theories of consciousness based on integrated information and causal density. However, these theories are based on network-theoretic descriptions of causal connectivity which are not well represented by considering only two regions. Further studies incorporating additional regions are therefore required to shed new light on the network-level dynamical changes underlying anaesthetic LOC. Such studies may, as a result, furnish novel theoretically-motivated procedures for assessing intraoperative anaesthetic depth [45] and for assaying residual consciousness in traumatically brain-injured patients [56]. The increased between-subject consistency we observed for GC, as compared to phase synchrony, further supports its potential use as a biomarker for consciousness.
